# Supplementary material for: Generating combinatorial diversity via engineered V(D)J-like recombination in Saccharomyces cerevisiae
Source: Nat Commun. 2025 Jul 1;16:5688. doi: 10.1038/s41467-025-61206-1 (PMC12216023; doi:10.1038/s41467-025-61206-1)
Supplement: Supplementary file 1 — Supplementary Information [file 41467_2025_61206_MOESM1_ESM.pdf]

SUPPLEMENTARY FIGURES AND TABLES

Generating combinatorial diversity via engineered V(D)J-like recombination in *Saccharomyces cerevisiae*

Andrew P. Cazier[1], Jaewoo Son[1], Sreenivas Yellayi[1], Lizmarie S. Chavez[1], Caden Young[1], Olivia M. Irvin[1], Hannah Abraham[1], Saachi Dalvi[1], and John Blazeck*[1]

[1]School of Chemical and Biomolecular Engineering, Georgia Institute of Technology, Atlanta GA 303332, USA.

Corresponding Author
John James Blazeck
Assistant Professor
john.blazeck@chbe.gatech.edu
School of Chemical and Biomolecular Engineering, Georgia Institute of Technology, Atlanta GA 303332, USA.

Table of Contents

Supplementary Figures

Figure S1. Confocal microscopy images of eGFP-tagged RAG proteins in NAB2-mCherry yeast SD2
Figure S2. Confocal microscopy images of eGFP-tagged RAG proteins in NOP56-mCherry yeast SD3
Figure S3. Figure S3. Protein sequence alignment of human and mouse HMGB1 .... SD4
Figure S4. Additional results from G418R recombination assay SD5
Figure S5. RAG1 truncation expression and correlation with recombination activity SD6
Figure S6. Additional protein characterization of G418R recombination SD7
Figure S7. Human RAG activity and expression SD8
Figure S8. Additional results from altering recombination target substrate SD9
Figure S9. Sequencing from integrated signal joint formation assay. SD10
Figure S10. Representative flow cytometry plots from GFP recombination SD11
Figure S11. Representative flow cytometry plots from scFv recombination SD12

Supplementary Tables

Table S1. Strains engineered in this work. SD13
Table S2. Synthesized gene sequences, yeast codon optimized SD16
Table S3. Recombination target and associated plasmids constructed in this work SD19
Table S4. RSS sequences SD21

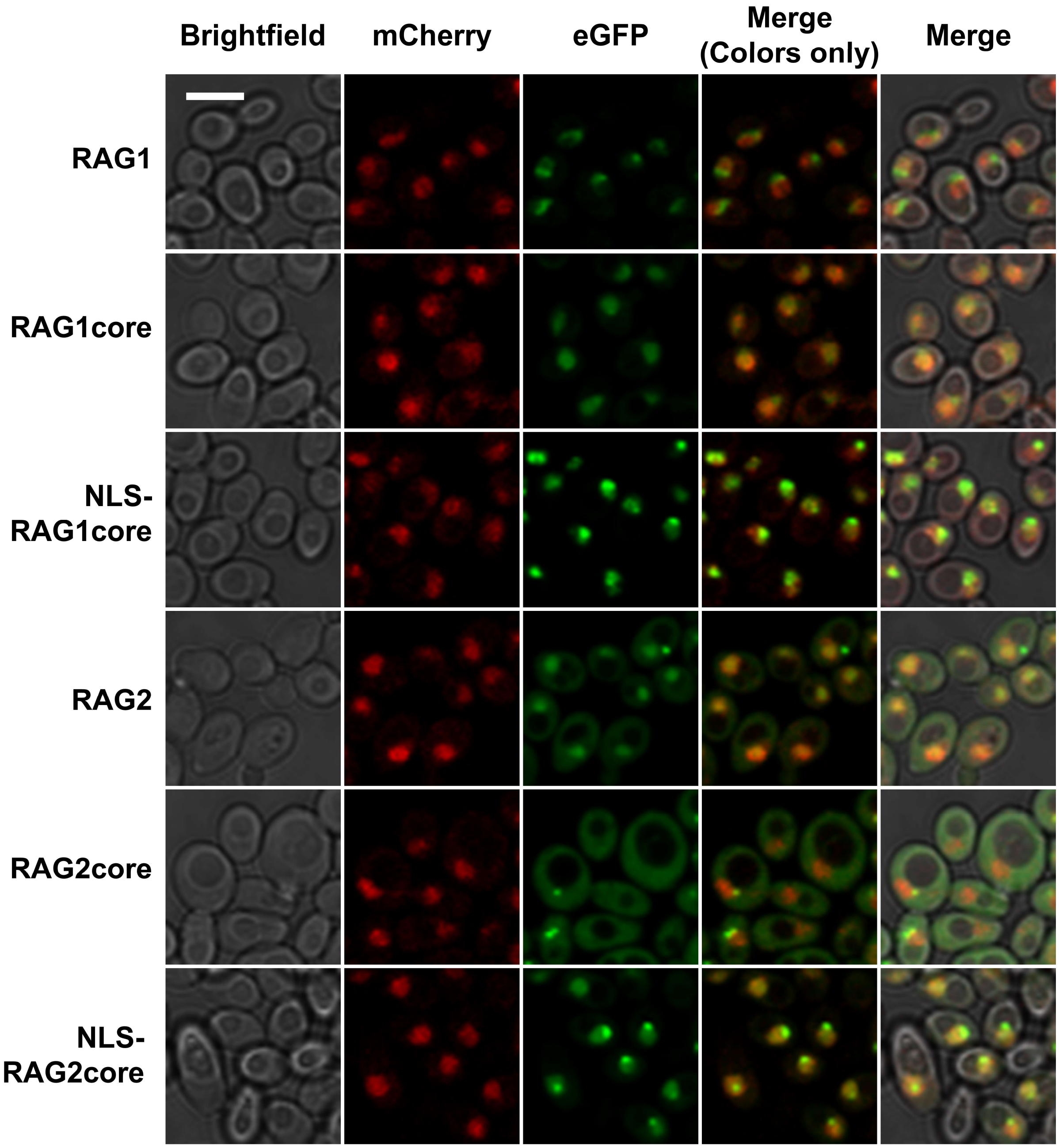

Figure S1. Confocal microscopy images of eGFP-tagged RAG proteins in NAB2-mCherry yeast. Cells were grown overnight in YPG prior to imaging on a Zeiss LSM-700 microscope. Each image set shows a subsection of a single z-stack slice. Images are representative of a group collected in biological triplicate (*n* = 3). Scale bar represents 5 μm; all images are at the same scale.

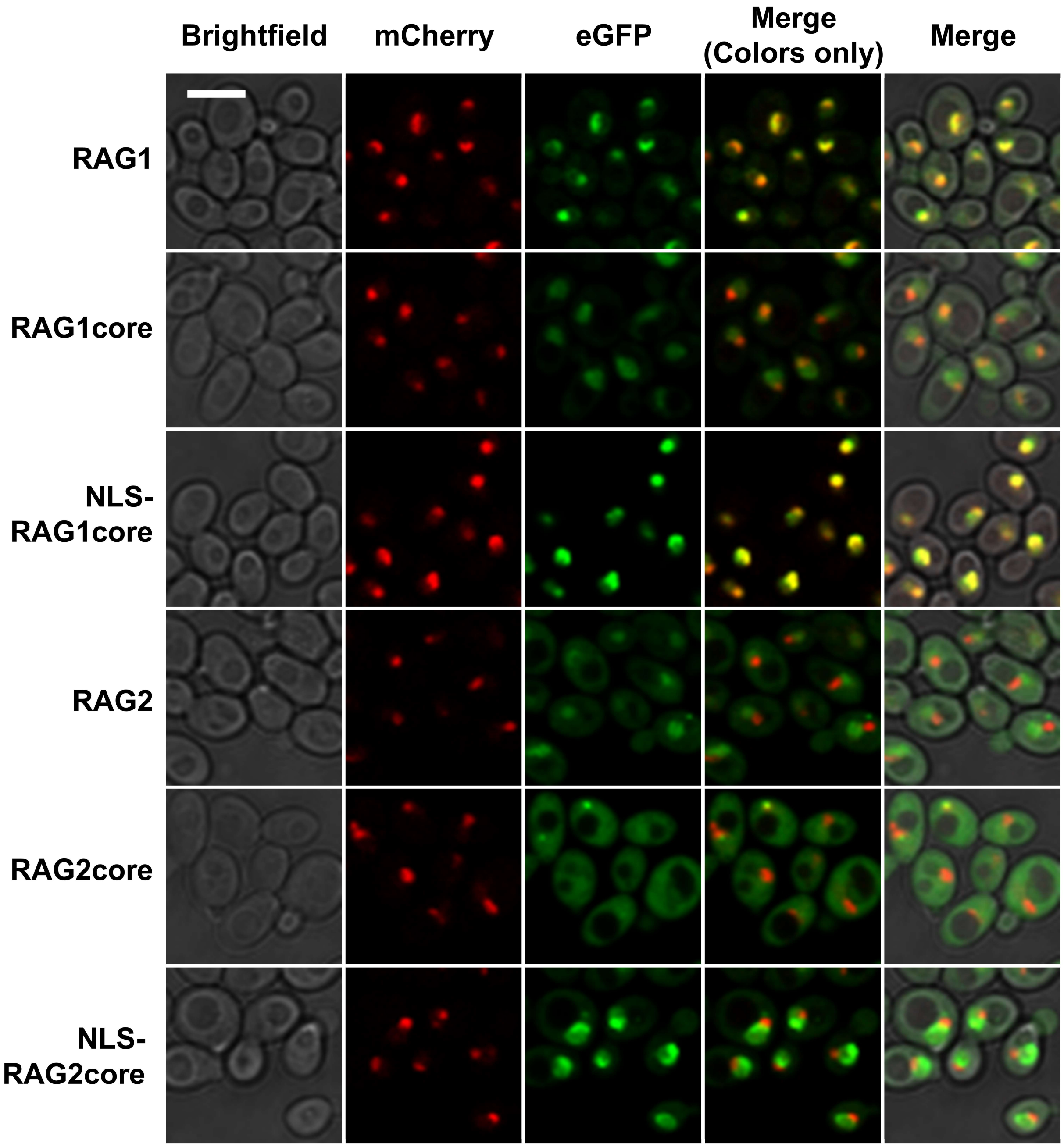

Figure S2. Confocal microscopy images of eGFP-tagged RAG proteins in NOP56-mCherry yeast. Cells were grown overnight in YPG prior to imaging on a Zeiss LSM-700 microscope. Each image set shows a subsection of a single z-stack slice. Images are representative of a group collected in biological triplicate (*n* = 3). Scale bar represents 5 µm; all images are at the same scale.

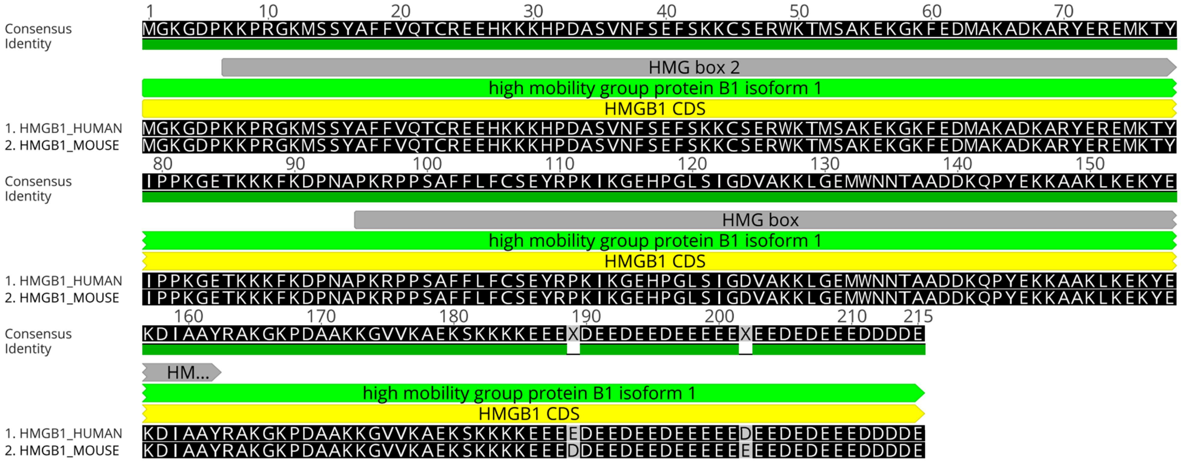

Figure S3. Protein sequence alignment of human and mouse HMGB1. The two sequences are nearly identical, sharing over 98% identity and differing only at two residues in the acidic tail domain.

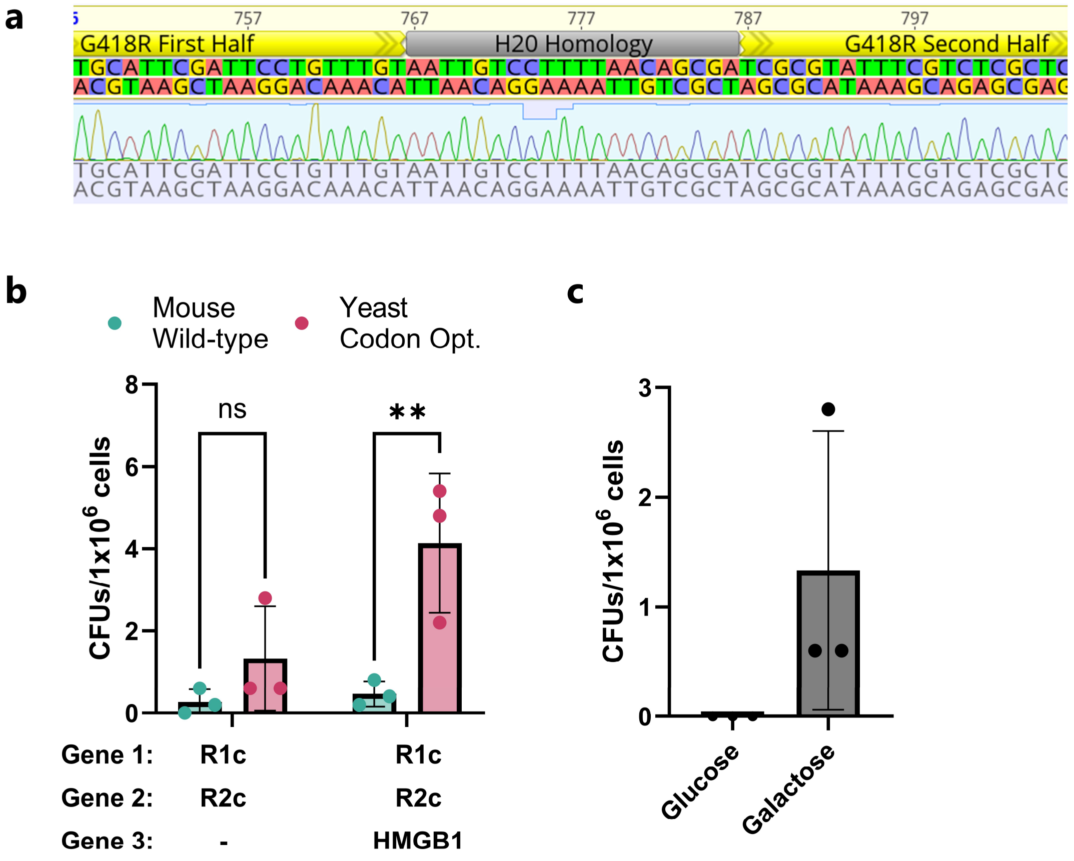

Figure S4. Additional results from G418R recombination assay. a) Representative sequencing of homology-assisted coding joint using pY112-CJA-UP-H20 target plasmid. **b**) G418R recombination assay to compare the effect of codon optimization. RAG1core and RAG2core were cloned in BY4742 yeast using either the wild-type mouse sequence or a yeast codon-optimized sequence. HMGB1 was always yeast codon optimized. **c**) G418R recombination assay to test the effect of induction media using a strain expressing RAG1core and RAG2core. As the RAG proteins were always expressed under GAL promoters, RAG expression will only occur when the yeast are cultured in galactose. CFU = colony forming unit. In b and c, data are presented as mean values ± SD; $n = 3$ biological replicates. In b, statistical significance was calculated with a two-way ANOVA and Fisher LSD test (ns = not significant, **$p<0.01$). From left to right, the highlighted p values in b are $p = 0.2623$ and 0.0032. Source data are provided in the Source Data file.

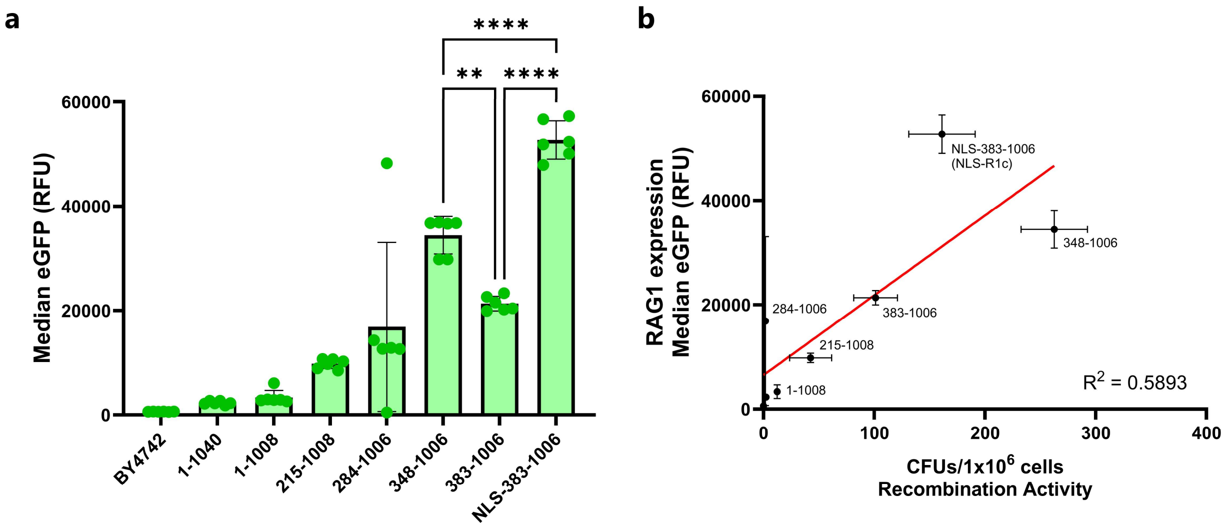

Figure S5. RAG1 truncation expression and correlation with recombination activity. **a**) Median eGFP fluorescence intensity of BY4742-NAB2-mCherry cells with RAG1 truncations tagged at the C-terminus with eGFP. Cells were induced overnight in YPG prior to analysis on a flow cytometer. Data are presented as mean ± SD; *n* = 6 biological replicates. **b**) Expression activity plotted versus recombination activity. CFU = colony forming unit. The y-axis data is taken from a, and the x-axis data is taken from Figure 2d. A line of best-fit along with the coefficient of determination, R^2, are displayed with the data. In a, statistical significance was calculated with a one-way ANOVA and Tukey test (**p=0.0054, ****p<0.0001). Source data are provided in the Source Data file.

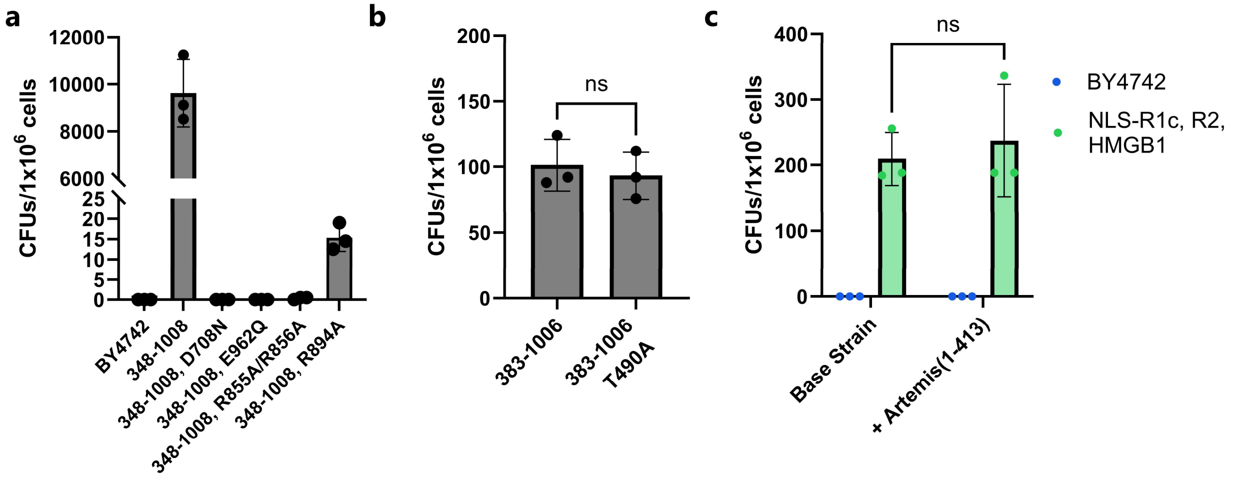

Figure S6. **Additional protein characterization of G418R recombination. a**) G418R recombination assay with pY112-CJA-U-H20 (plasmid described in **Figure 3**). All recombination strains had RAG1(348-1008) (or a mutant thereof), RAG2, and HMGB1. D708N and E962Q are RAG1 mutations of essential catalytic residues. R855A/R856A and R894A are RAG1 mutations which have impaired hairpin formation but can still nick DNA. BY4742 and 348-1008 are repeated from Figure 3f; both figures are derived from the same experiment. **b**) G418R recombination assay with pY112-CJA-UP-H20 to test the effect of the T490A mutation in RAG2. Both strains had RAG1core, HMGB1, and either normal RAG2 (left) or RAG2-T490A (right). 348-1006 is repeated from Figure 2d as both figures are derived from the same experiment. **c**) G418R recombination assay with pY112-CJA-UP-H20 comparing the effect of adding a truncated human Artemis protein to a base strain, BY4742, and a recombination-competent strain containing NLS-RAG1core, RAG2, HMGB1. CFU = colony forming unit. For a, b, and c, cells were plated after a 4d induction; data are presented as mean values ± SD; n = 3 biological replicates. In b, statistical significance was calculated with an unpaired two-sided t-test, p = 0.9997, and in c, statistical significance was calculated with a two-way ANOVA and Tukey test, p = 0.4889 (ns = not significant). Source data are provided in the Source Data file.

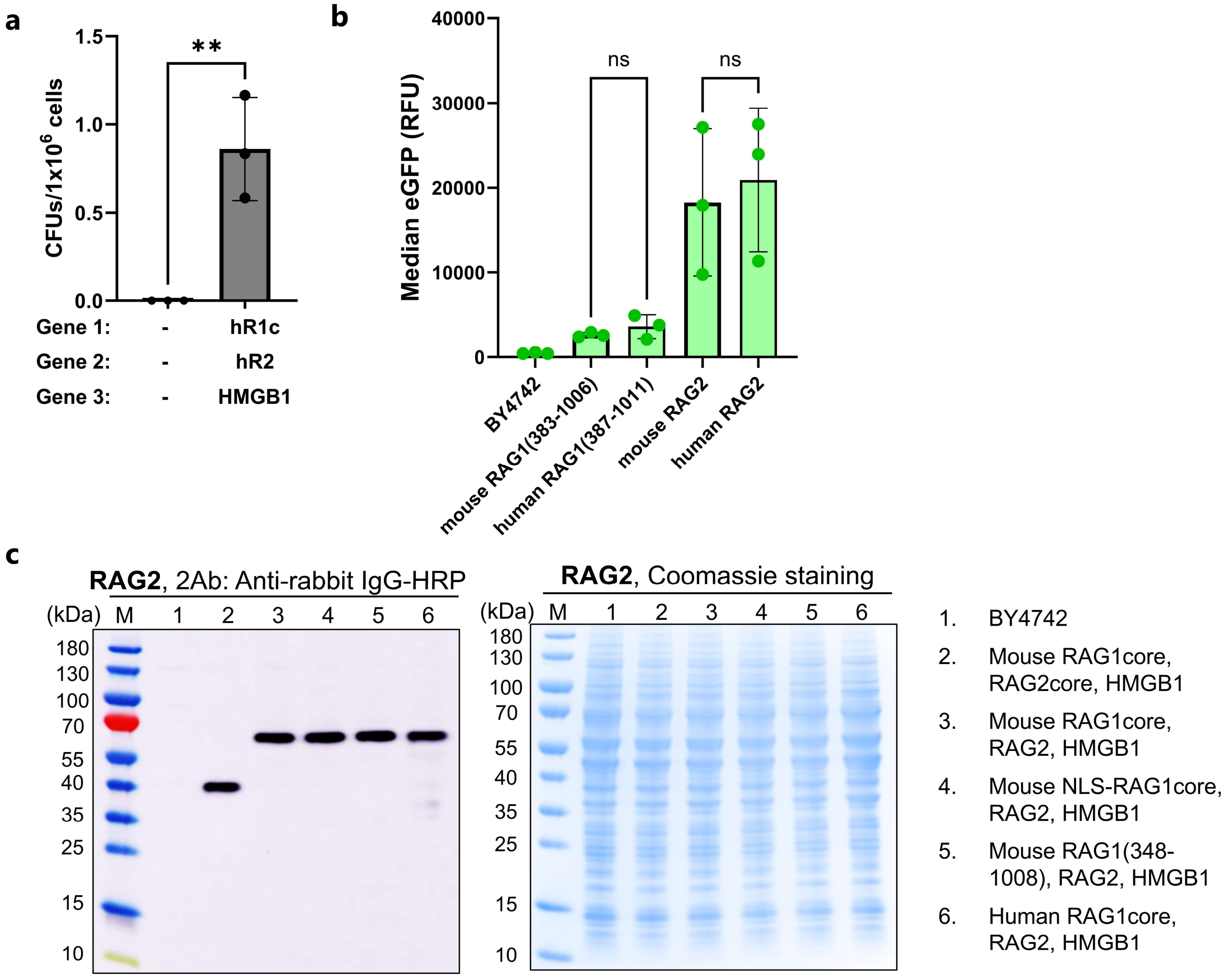

Figure S7. Human RAG activity and expression. **a**) G418R recombination test with pY112-CJA-UP-H20 plasmid after 4d using human RAG1core, human RAG2, and HMGB1 relative to wild-type BY4742 cells. CFU = colony forming unit. **b**) Human RAG1core or RAG2 were tagged with eGFP on the C-terminus and integrated in BY4742-NAB2-mCherry yeast and compared to the equivalent mouse proteins tagged with eGFP. Median eGFP fluorescence was then measured using flow cytometry after overnight culture in YPG. **c**) Western blot of RAG2 or RAG2core in protein extracted from various recombination strains (left panel). Protein loading was verified by a separate gel stained with Coomassie Blue (right panel). In a and b, data are presented as mean values ± SD; *n* = 3 biological replicates. In a, statistical significance was calculated with an unpaired two-sided t-test, p = 0.0070, and in b, statistical significance was calculated with a one-way ANOVA and Tukey test; from left to right, p = 0.9994 and 0.9729 (ns = not significant, **p<0.01). Source data are provided in the Source Data file.

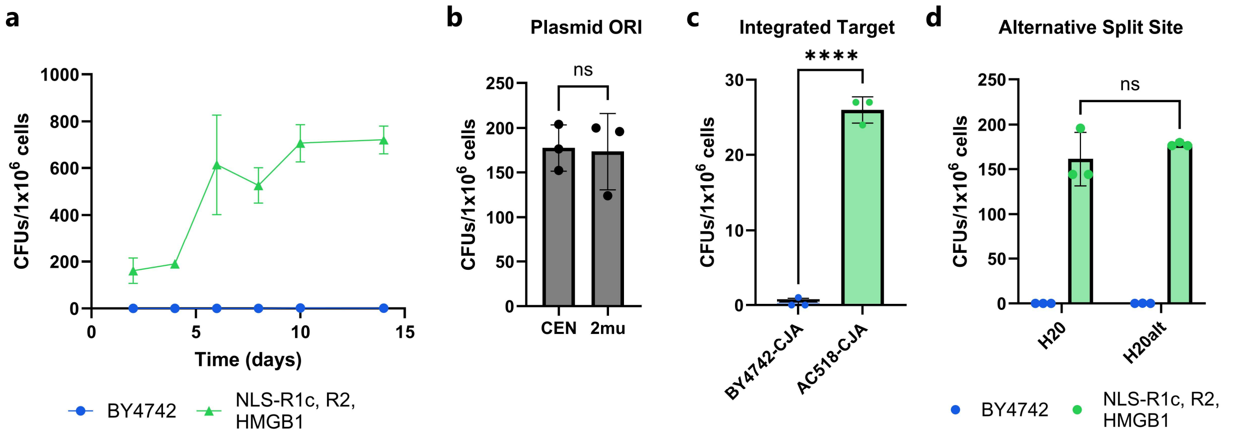

Figure S8. Additional results from altering recombination target substrate. a) Time course of G418 resistant colony accumulation using the pY112-CJA-UP-H20 target plasmid in the specified strains. Cells were cultured in SG-Leu for 14d total and plated at the specified timepoints. **b**) G418R recombination in strain AC518 (NLS-RAG1core, RAG2, and HMGB1) comparing the effect of plasmid origin of replication (and copy number) on recombination using the "UP" spacer and 20 bp of homology. Cells were plated after a 4d induction. **c**) G418R recombination using the "UP" spacer and 20 bp of homology that has been integrated into the genome of AC518 or BY4742. Cells were plated after a 4d induction in SG media prior to plating on YPD G418 plates. **d**) G418R recombination assay using the pY112-CJA-UP-H20 target versus pY112-CJA-UP-H20alt. In the "alt" plasmid, G418R was split in a different location, and thus has a different 20 bp of homology relative to the standard target. Cells were plated after 4d induction. CFU = colony forming unit. For a-d, data are presented as mean values ± SD; *n* = 3 biological replicates. In b and c, statistical significance was calculated using an unpaired two-sided t-test; in b, p = 0.8966, and in c, p = <0.0001. In d, significance was calculated using a two-way ANOVA and Tukey test, p = 0.2293 (ns = not significant, ****p<0.0001). Source data are provided in the Source Data file.

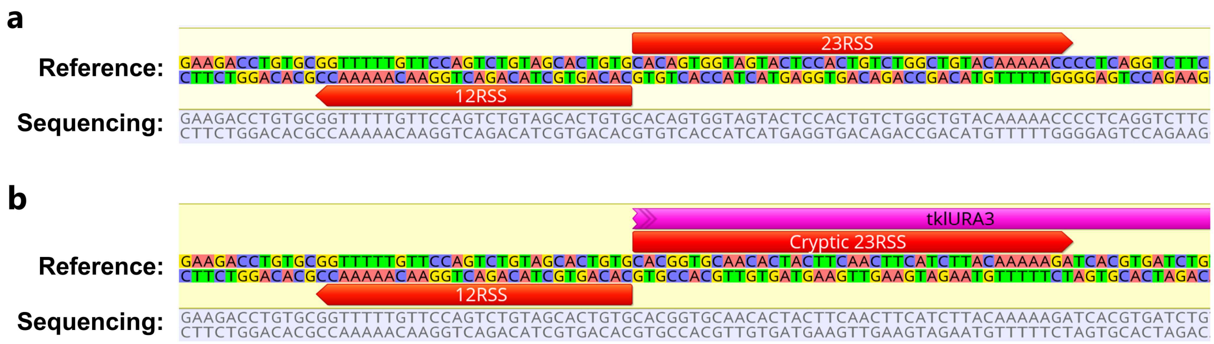

Figure S9. Sequencing from integrated signal joint formation assay. a) Sequencing of a non-fluorescent, AC518-SJ colony grown on a 5-FOA plate. The RSSs are joined without error at the heptamer, forming a canonical signal joint. **b**) Sequencing of a fluorescent, AC518-SJ colony grown on a 5-FOA plate. The cryptic RSS heptamer differs at only one position from the consensus sequence, and the nonamer only differs at two nucleotides.

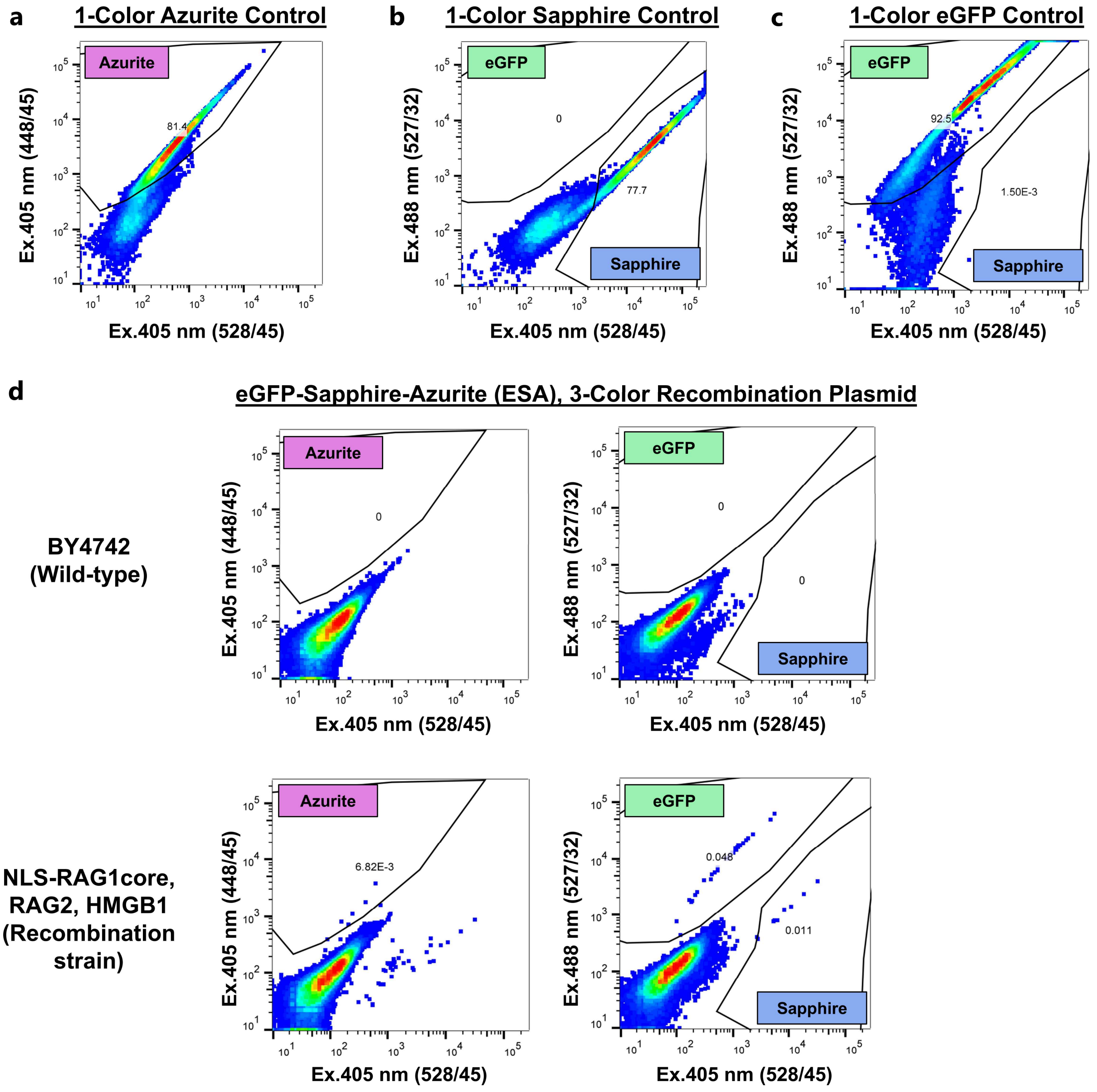

Figure S10. Representative flow cytometry plots from GFP recombination. **a**) Single-color control showing how Azurite events were gated using two channels. **b**) Single-color control showing how Sapphire events were gated using two channels. **c**) Single-color control showing how eGFP events were gated using two channels. **d**) Three-color recombination with the pY112-CJCG-ESA-H20 plasmid. BY4742 are wild-type cells that do not contain V(D)J recombination genes. Cells were induced in SG-Leu media for 9d prior to flow cytometry.

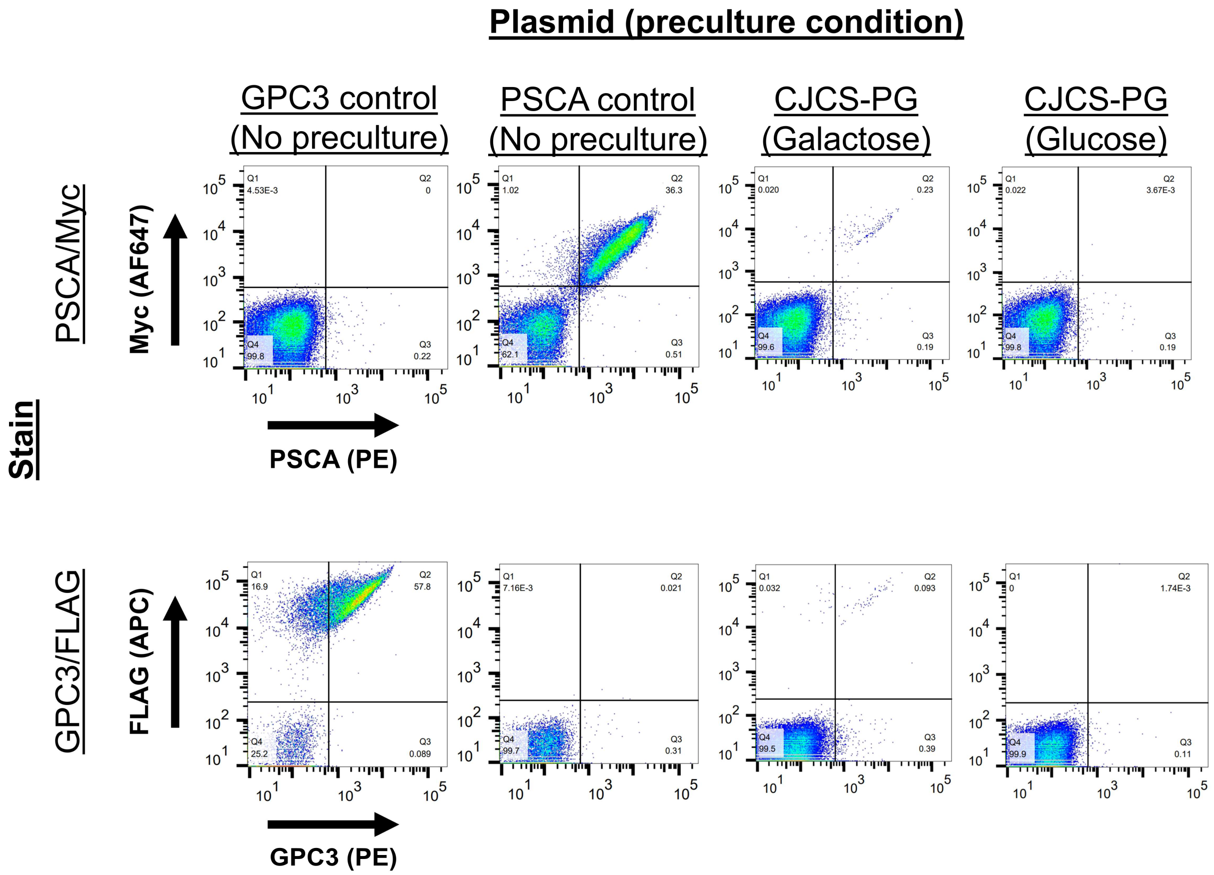

Figure S11. **Representative flow cytometry plots from scFv recombination**. Each condition was treated with two separate stains, one for PSCA/Myc and another for GPC3/FLAG. In each plot, the x-axis corresponds to antigen binding and the y-axis to peptide presentation. All plots are from strain AC701 which contains NLS-RAG1core, RAG2, and HMGB1. Cells with pY110-CJCS-PG plasmid were precultured in either SG-Trp or SD-Trp media for 8d. Then all cells, including single-scFv controls, were grown in SD-Trp for 1d followed by buffered SG-Trp for 1d to induce display. All plots were derived from forward scatter (FSC) singlet populations.

Table S1. Strains engineered in this work

Strain	Base	Modifications to Base Strain
BY4742-NAB2	*BY4742*	YPRCΔ15::pNAB2-NAB2-mCherry-tADH1, psmTEF1-snNAT-tCYC1
BY4742-NOP56	*BY4742*	YPRCΔ15::pNOP56-NOP56-mCherry-tADH1, psmTEF1-snNAT-tCYC1
AC411	*BY4742-NAB2*	YORWΔ22::pGAL1-RAG1-eGFP-tPRM9, pagTEF1-klLEU2-tagTEF1
AC412	*BY4742-NAB2*	YORWΔ22::pGAL1-RAG1core-eGFP-tPRM9, pagTEF1-klLEU2-tagTEF1
AC413	*BY4742-NAB2*	YORWΔ22::pGAL1-hht1NLS-RAG1core-eGFP-tPRM9, pagTEF1-klLEU2-tagTEF1
AC414	*BY4742-NAB2*	YORWΔ22::pGAL1-RAG2-eGFP-tPRM9, pagTEF1-klLEU2-tagTEF1
AC415	*BY4742-NAB2*	YORWΔ22::pGAL1-RAG2core-eGFP-tPRM9, pagTEF1-klLEU2-tagTEF1
AC416	*BY4742-NAB2*	YORWΔ22::pGAL1-hht1NLS-RAG2core-eGFP-tPRM9, pagTEF1-klLEU2-tagTEF1
AC421	*BY4742-NOP56*	YORWΔ22::pGAL1-RAG1-eGFP-tPRM9, pagTEF1-klLEU2-tagTEF1
AC422	*BY4742-NOP56*	YORWΔ22::pGAL1-RAG1core-eGFP-tPRM9, pagTEF1-klLEU2-tagTEF1
AC423	*BY4742-NOP56*	YORWΔ22::pGAL1-hht1NLS-RAG1core-eGFP-tPRM9, pagTEF1-klLEU2-tagTEF1
AC424	*BY4742-NOP56*	YORWΔ22::pGAL1-RAG2-eGFP-tPRM9, pagTEF1-klLEU2-tagTEF1
AC425	*BY4742-NOP56*	YORWΔ22::pGAL1-RAG2core-eGFP-tPRM9, pagTEF1-klLEU2-tagTEF1
AC426	*BY4742-NOP56*	YORWΔ22::pGAL1-hht1NLS-RAG2core-eGFP-tPRM9, pagTEF1-klLEU2-tagTEF1
AC427	*BY4742-NAB2*	YORWΔ22::pGAL1-RAG1(1-1008)-eGFP-tPRM9, pagTEF1-klLEU2-tagTEF1
AC428	*BY4742-NAB2*	YORWΔ22::pGAL1-RAG1(215-1008)-eGFP-tPRM9, pagTEF1-klLEU2-tagTEF1
AC429	*BY4742-NAB2*	YORWΔ22::pGAL1-RAG1(284-1006)-eGFP-tPRM9, pagTEF1-klLEU2-tagTEF1
AC430	*BY4742-NAB2*	YORWΔ22::pGAL1-RAG1(348-1006)-eGFP-tPRM9, pagTEF1-klLEU2-tagTEF1
AC431	*BY4742-NAB2*	YORWΔ22::pGAL1-hht1NLS-RAG1core-eGFP-tPRM9, pagTEF1-klLEU2-tagTEF1
AC432	*BY4742-NAB2*	YORWΔ22::pGAL1-hsRAG1(387-1011)-eGFP-tPRM9, pagTEF1-klLEU2-tagTEF1
AC433	*BY4742-NAB2*	YORWΔ22::pGAL2-hsRAG2-eGFP-tPRM9, pagTEF1-klLEU2-tagTEF1
AC434	*BY4742-NAB2*	YORWΔ22::pGAL2-RAG2-eGFP-tPRM9, pagTEF1-klLEU2-tagTEF1

AC501	*BY4742*	YPRCτ3::pGAL1-RAG1-tPRM9, pGAL2-RAG2-tRPL15A, psmTEF1-snNAT-tagTEF1
AC502	*BY4742*	YPRCτ3::pGAL1-RAG1-tPRM9, pGAL2-RAG2-tRPL15A, psmTEF1-snNAT-tagTEF1, pspTDH3-HMGB1-tRPL41B
AC503	*BY4742*	YPRCτ3::pGAL1-RAG1core-tPRM9, pGAL2-RAG2core-tRPL15A, psmTEF1-snNAT-tagTEF1
AC504	*BY4742*	YPRCτ3::pGAL1-RAG1core-tPRM9, pGAL2-RAG2core-tRPL15A, psmTEF1-snNAT-tagTEF1, pspTDH3-HMGB1-tRPL41B
AC505	*BY4742*	YPRCτ3::pGAL1-RAG1core-tPRM9, pGAL2-RAG2-tRPL15A, psmTEF1-snNAT-tagTEF1
AC506	*BY4742*	YPRCτ3::pGAL1-RAG1core-tPRM9, pGAL2-RAG2-tRPL15A, psmTEF1-snNAT-tagTEF1, pspTDH3-HMGB1-tRPL41B
AC507	*BY4742*	YPRCτ3::pGAL1-RAG1core-tPRM9, pGAL2-hht1NLS-RAG2core-tRPL15A, psmTEF1-snNAT-tagTEF1
AC508	*BY4742*	YPRCτ3::pGAL1-hht1NLS-RAG1core-tPRM9, pGAL2-hht1NLS-RAG2core-tRPL15A, psmTEF1-snNAT-tagTEF1
AC509	*BY4742*	YPRCτ3::pGAL1-hht1NLS-RAG1core-tPRM9, pGAL2-hht1NLS-RAG2core-tRPL15A, psmTEF1-snNAT-tagTEF1, pspTDH3-HMGB1-tRPL41B
AC510	*BY4742*	YPRCτ3::pGAL1-wtRAG1core-tPRM9, pGAL2-wtRAG2core-tRPL15A, psmTEF1-snNAT-tagTEF1
AC511	*BY4742*	YPRCτ3::pGAL1-wtRAG1core-tPRM9, pGAL2-wtRAG2core-tRPL15A, psmTEF1-snNAT-tagTEF1, pspTDH3-HMGB1-tRPL41B
AC512	*BY4742*	YPRCτ3::pGAL1-hsRAG1(387-1011)-tPRM9, pGAL2-hsRAG2-tRPL15A, psmTEF1-snNAT-tagTEF1, pspTDH3-HMGB1-tRPL41B
AC513	*BY4742*	YPRCτ3::pGAL1-RAG1(1-1008)-tPRM9, pGAL2-RAG2-tRPL15A, psmTEF1-snNAT-tagTEF1, pspTDH3-HMGB1-tRPL41B
AC514	*BY4742*	YPRCτ3::pGAL1-RAG1(215-1008)-tPRM9, pGAL2-RAG2-tRPL15A, psmTEF1-snNAT-tagTEF1, pspTDH3-HMGB1-tRPL41B
AC515	*BY4742*	YPRCτ3::pGAL1-RAG1(284-1006)-tPRM9, pGAL2-RAG2-tRPL15A, psmTEF1-snNAT-tagTEF1, pspTDH3-HMGB1-tRPL41B
AC516	*BY4742*	YPRCτ3::pGAL1-RAG1(348-1006)-tPRM9, pGAL2-RAG2-tRPL15A, psmTEF1-snNAT-tagTEF1, pspTDH3-HMGB1-tRPL41B
AC517	*BY4742*	YPRCτ3::pGAL1-RAG1core-tPRM9, pGAL2-RAG2(T490A)-tRPL15A, psmTEF1-snNAT-tagTEF1, pspTDH3-HMGB1-tRPL41B
AC518	*BY4742*	YPRCτ3::pGAL1-hht1NLS-RAG1core-tPRM9, pGAL2-RAG2-tRPL15A, psmTEF1-snNAT-tagTEF1, pspTDH3-HMGB1-tRPL41B
AC519	*BY4742*	YPRCτ3::pGAL1-RAG1(348-1008)-tPRM9, pGAL2-RAG2-tRPL15A, psmTEF1-snNAT-tagTEF1, pspTDH3-HMGB1-tRPL41B
AC520	*BY4742*	YPRCτ3::pGAL1-RAG1(348-1008)D708N -tPRM9, pGAL2-RAG2-tRPL15A, psmTEF1-snNAT-tagTEF1, pspTDH3-HMGB1-tRPL41B
AC521	*BY4742*	YPRCτ3::pGAL1-RAG1(348-1008)E962Q-tPRM9, pGAL2-RAG2-tRPL15A, psmTEF1-snNAT-tagTEF1, pspTDH3-HMGB1-tRPL41B
AC522	*BY4742*	YPRCτ3::pGAL1-RAG1(348-1008)R855A/R856A -tPRM9, pGAL2-RAG2-tRPL15A, psmTEF1-snNAT-tagTEF1, pspTDH3-HMGB1-tRPL41B

AC523	*BY4742*	YPRCτ3::pGAL1-RAG1(348-1008)R894A-tPRM9, pGAL2-RAG2-tRPL15A, psmTEF1-snNAT-tagTEF1, pspTDH3-HMGB1-tRPL41B
AC518-SJ	*AC518*	NRT1/GYP1::SJUEout
BY4742-SJ	*BY4742*	NRT1/GYP1::SJUEout
AC518-CJA	*AC518*	NRT1/GYP1::CJA-UP-H20
BY4742-CJA	*BY4742*	NRT1/GYP1::CJA-UP-H20
AC518-Art413	*AC518*	YORWΔ22::pTEF1-hph-tagTEF1, pFBA1-hsArtemis(1-413)-tVMA2
BY4742-Art413	*BY4742*	YORWΔ22::pTEF1-hph-tagTEF1, pFBA1-hsArtemis(1-413)-tVMA2
AC601	*BY4742*	YPRCτ3::pGAL1-RAG1core(T400P)-tPRM9, pGAL2-RAG2-tRPL15A, psmTEF1-snNAT-tagTEF1, pspTDH3-HMGB1-tRPL41B
AC602	*BY4742*	YPRCτ3::pGAL1-RAG1core(G513A)-tPRM9, pGAL2-RAG2-tRPL15A, psmTEF1-snNAT-tagTEF1, pspTDH3-HMGB1-tRPL41B
AC603	*BY4742*	YPRCτ3::pGAL1-RAG1core(R556S)-tPRM9, pGAL2-RAG2-tRPL15A, psmTEF1-snNAT-tagTEF1, pspTDH3-HMGB1-tRPL41B
AC604	*BY4742*	YPRCτ3::pGAL1-RAG1core(R696Q)-tPRM9, pGAL2-RAG2-tRPL15A, psmTEF1-snNAT-tagTEF1, pspTDH3-HMGB1-tRPL41B
AC605	*BY4742*	YPRCτ3::pGAL1-RAG1core(K989E)-tPRM9, pGAL2-RAG2-tRPL15A, psmTEF1-snNAT-tagTEF1, pspTDH3-HMGB1-tRPL41B
AC701	*EBY100*	YPRCτ3::pGAL1-hht1NLS-RAG1core-tPRM9, pGAL2-RAG2-tRPL15A, psmTEF1-snNAT-tagTEF1, pspTDH3-HMGB1-tRPL41B

Note: All promoters and terminators are from *S. cerevisiae* unless otherwise noted. All RAG genes are yeast-codon-optimized mouse sequences except where noted. RAG1core includes residues 383-1006 and RAG2core includes residues 1-383. Abbreviations: ag = *Ashbya gossypii*, *hs = Homo sapiens*, *kl = Kluyveromyces lactis*, nc = *Naumovozyma castellii*, NLS = nuclear localization signal, sm = *Saccharomyces mikatae*, sn = *Streptomyces noursei*, wt = wild-type sequence

Table S2. Synthesized gene sequences, yeast codon optimized

```
>RAG1_mouse
ATGGCTGCCTCCTTGCCGTCTACCTTGAGCTTCAGTTCTGCACCCGATGAAATTCAACATCCACAAATCAAATTTTCCGAGTG
GAAATTTAAGCTGTTTAGGGTTAGATCCTTTGAAAAGGCACCCGAAGAAGCACAAAAGGAAAAAGATTCCTCAGAAGGGAAAC
CTTACCTAGAACAATCTCCAGTAGTTCCAGAGAAGCCTGGTGGCCAAAACTCAATTTTGACTCAACGTGCATTGAAATTGCAT
CCTAAATTTTCAAAGAAATTTCATGCTGATGGGAAGTCAAGCGACAAAGCAGTTCACCAAGCCAGGTTGAGACATTTCTGTAG
AATCTGTGGTAATCGTTTCAAGAGTGACGGTCACTCAAGAAGATACCCAGTTCATGGTCCCGTTGACGCTAAAACCCAAAGTC
TTTTCCGTAAAAAGGAAAAAAGAGTAACTTCCTGGCCAGACTTGATTGCCAGGATTTTCAGAATCGACGTAAAAGCAGATGTT
GACTCCATTCACCCGACGGAATTCTGCCATGATTGTTGGAGCATCATGCATAGAAAGTTCTCTAGTTCTCACAGTCAGGTTTA
CTTCCCAAGGAAAGTAACTGTGGAGTGGCACCCCCACACACCGTCCTGTGACATTTGTTTTACTGCACATAGAGGATTGAAGA
GGAAGAGACATCAGCCCAATGTACAGCTATCTAAGAAACTAAAAACTGTGTTGAACCACGCGAGAAGGGATCGTAGAAAGAGA
ACTCAAGCTAGGGTCAGTTCAAAGGAAGTCCTAAAGAAGATCTCCAACTGCAGTAAGATTCATTTGAGTACAAAGCTTCTTGC
CGTGGACTTCCCAGCACACTTTGTGAAATCCATCTCCTGCCAAATATGCGAACACATTTTAGCTGATCCCGTTGAGACAAGCT
GCAAGCATCTATTCTGTCGTATTTGCATTCTGAGATGTTTAAAAGTTATGGGCTCTTATTGTCCCTCTTGCAGGTATCCGTGC
TTCCCTACTGACTTAGAAAGCCCAGTTAAGTCCTTTTTAAACATTTTGAATTCTCTGATGGTTAAGTGTCCCGCGCAAGATTG
CAATGAGGAAGTGAGTTTAGAAAAATATAACCACCATGTTTCAAGTCACAAAGAATCTAAAGAGACTTTGGTTCATATCAATA
AAGGTGGAAGACCTAGACAACATCTACTGTCATTAACGAGAAGGGCGCAGAAACATAGATTAAGGGAGTTAAAGATTCAAGTT
AAAGAATTTGCTGACAAAGAAGAAGGTGGGGATGTAAAAGCTGTTTGCTTGACATTGTTTCTACTGGCACTGAGGGCGAGGAA
TGAACACAGGCAAGCTGATGAATTAGAGGCCATAATGCAAGGCAGGGGATCCGGTCTTCAACCAGCTGTTTGCTTGGCTATCC
GTGTCAATACTTTTCTTAGCTGTTCTCAATATCATAAGATGTATAGGACTGTTAAAGCTATAACTGGTAGGCAAATTTTTCAA
CCTTTGCATGCTCTTAGAAATGCCGAAAAAGTTCTTTTACCAGGTTACCATCCATTTGAGTGGCAACCTCCATTGAAGAATGT
TTCCTCTAGAACTGATGTTGGAATAATTGATGGTCTGTCTGGATTGGCATCTTCTGTTGATGAATATCCAGTAGATACGATTG
CGAAAAGGTTCAGATACGATTCTGCTTTGGTTTCTGCTTTGATGGATATGGAAGAAGACATCTTGGAAGGCATGAGATCCCAA
GATCTTGATGACTACTTGAATGGTCCATTTACAGTTGTTGTAAAGGAATCTTGCGATGGAATGGGAGATGTGAGTGAAAAGCA
CGGAAGTGGGCCTGCAGTTCCAGAAAAGGCGGTTCGTTTCTCTTTCACAGTTATGAGAATTACGATAGAACATGGTTCACAAA
ACGTGAAGGTTTTTGAGGAACCCAAGCCTAATTCTGAATTATGTTGCAAGCCGTTGTGTCTTATGTTGGCAGATGAGTCTGAC
CATGAGACACTTACTGCTATTCTAAGCCCCCTAATTGCTGAGAGGGAAGCCATGAAGAGTAGTGAATTAACGCTAGAGATGGG
AGGCATCCCGAGGACTTTTAAATTCATATTCAGGGGTACTGGATACGATGAAAAACTTGTCAGGGAAGTAGAAGGTTTGGAAG
CTTCTGGCTCAGTATATATCTGTACATTGTGTGATACCACCCGTTTGGAAGCCTCTCAAAATCTTGTCTTCCACTCCATAACG
AGAAGCCACGCAGAGAACCTGCAGAGATATGAGGTCTGGAGATCAAATCCGTATCATGAGTCCGTGGAAGAGTTGCGTGACAG
AGTGAAAGGTGTCTCTGCCAAACCTTTCATCGAAACAGTTCCTTCAATAGATGCGCTTCACTGTGATATTGGTAATGCAGCTG
AATTTTATAAGATTTTCCAGCTGGAGATAGGTGAAGTATATAAACATCCTAATGCCTCTAAAGAAGAAAGAAAGAGATGGCAA
GCCACGTTAGATAAACATCTTAGGAAAAGAATGAACTTAAAACCAATCATGAGGATGAATGGTAACTTTGCCAGAAAGTTGAT
GACTCAAGAGACTGTAGATGCAGTTTGTGAATTAATTCCTTCTGAGGAGAGGCATGAAGCTTTGAGGGAGTTGATGGACTTAT
ATCTAAAAATGAAACCAGTGTGGAGATCTTCATGTCCAGCTAAAGAATGTCCAGAGAGTTTGTGTCAATACAGTTTCAACTCA
CAGCGTTTCGCGGAACTACTGAGTACCAAGTTTAAATATAGATATGAAGGTAAAATAACTAATTACTTTCATAAAACCTTGGC
ACATGTCCCTGAAATTATTGAAAGGGATGGTTCTATCGGGGCATGGGCAAGTGAAGGAAATGAAAGCGGTAACAAATTGTTTA
GAAGATTTAGGAAAATGAATGCCAGGCAGTCTAAGTGCTATGAGATGGAAGATGTCCTGAAACATCACTGGTTGTATACTTCA
AAATACTTACAGAAGTTTATGAATGCTCATAACGCGTTAAAAAGCTCTGGATTTACCATGAACTCAAAGGAGACATTAGGGGA
CCCTTTGGGAATTGAGGATTCTCTGGAAAGCCAAGATTCAATGGAGTTTTAA
```

```
>RAG2_mouse
ATGTCCCTGCAGATGGTAACAGTGGGTCATAACATAGCGTTAATTCAACCAGGTTTCTCACTTATGAATTTTGATGGCCAAGT
TTTCTTCTTTGGCCAAAAAGGCTGGCCAAAGCGTTCCTGTCCTACTGGTGTCTTTCATTTTGATATAAAACAAAATCATTTAA
AACTGAAGCCTGCAATCTTCTCTAAAGATTCCTGCTACCTACCACCTTTGCGTTACCCAGCTACTTGCTCATACAAAGGTAGC
ATAGACTCTGACAAGCATCAATATATCATTCACGGTGGGAAGACACCGAACAATGAGCTATCCGATAAGATTTATATCATGTC
TGTTGCTTGCAAGAATAACAAAAAAGTTACTTTCCGTTGCACAGAGAAAGACTTAGTAGGTGATGTCCCTGAACCAAGATACG
GCCATAGCATTGACGTGGTGTATAGTAGAGGTAAAAGCATGGGTGTTCTGTTTGGAGGTAGGTCATACATGCCTTCTACCCAA
CGTACCACAGAAAAATGGAATAGTGTAGCTGACTGTCTACCCCATGTTTTCTTGATAGATTTTGAATTTGGTTGTGCTACATC
ATATATTTTGCCAGAACTTCAAGATGGGCTGTCTTTTCATGTTTCTATTGCCAGAAACGATACGGTTTATATTTTGGGGGGAC
ACTCATTAGCCAGTAATATAAGACCTGCTAACTTGTATCGTATAAGAGTGGACCTTCCACTGGGTACCCCAGCAGTGAATTGT
ACAGTCTTGCCAGGAGGAATCTCTGTATCCAGTGCGATCTTAACTCAAACAAACAATGATGAATTTGTTATTGTGGGTGGTTA
TCAGCTGGAAAATCAGAAAAGGATGGTCTGTAGCTTGGTTTCTCTAGGTGACAACACGATTGAAATCAGTGAGATGGAGACTC
CAGACTGGACGTCAGATATTAAGCATAGCAAAATATGGTTTGGAAGCAACATGGGAAACGGTACTATTTTCCTTGGCATACCA
GGAGACAATAAGCAGGCTATGTCAGAAGCATTCTATTTCTATACTTTGAGATGCTCTGAAGAGGATTTGAGTGAAGATCAGAA
AATTGTCTCCAACAGTCAAACATCAACAGAAGATCCTGGTGACTCCACTCCCTTTGAAGACTCAGAGGAATTTTGTTTCAGTG
```

CTGAAGCAACCAGTTTTGATGGTGACGATGAATTTGACACCTACAATGAAGATGATGAAGATGACGAGTCTGTAACCGGCTAC
TGGATAACATGTTGTCCGACTTGTGATGTTGACATCAATACCTGGGTTCCGTTCTATTCAACGGAGTTAAATAAACCAGCCAT
GATCTATTGTTCTCATGGTGATGGGCACTGGGTACATGCCCAATGCATGGATTTGGAAGAAAGAACATTGATCCACTTGTCAG
AAGGAAGCAACAAGTATTATTGCAATGAACATGTACAAATAGCAAGAGCATTGCAAACTCCCAAAAGAAACCCCCCCTTACAA
AAACCTCCAATGAAATCCTTGCACAAAAAAGGCTCTGGTAAAGTCTTGACTCCTGCCAAGAAATCCTTCCTTAGAAGACTGTT
TGATTAA

>HMGB1_human
ATGGGAAAAGGAGATCCAAAAAAGCCTAGAGGCAAGATGTCATCTTATGCATTTTTCGTTCAAACATGCAGAGAAGAACACAA
GAAAAAACACCCAGACGCCTCAGTGAATTTTTCTGAATTCTCAAAAAAATGTTCAGAAAGGTGGAAAACTATGAGTGCCAAAG
AAAAGGGTAAATTTGAGGACATGGCAAAAGCCGATAAGGCAAGATACGAAAGAGAAATGAAAACTTACATTCCACCAAAGGGA
GAAACCAAAAAGAAATTCAAGGACCCCAATGCACCTAAGAGGCCCCCATCCGCATTTTTCTTGTTTTGTTCCGAATATCGTCC
TAAAATAAAAGGTGAACATCCTGGTTTGTCTATAGGTGACGTTGCAAAGAAGTTAGGAGAAATGTGGAACAACACTGCTGCTG
ATGATAAACAGCCTTACGAAAAGAAAGCAGCCAAACTAAAAGAAAAATATGAGAAGGATATCGCAGCATACAGGGCTAAAGGT
AAACCCGATGCTGCAAAGAAAGGAGTAGTCAAGGCTGAAAAATCCAAGAAAAAGAAGGAGGAGGAAGAAGATGAAGAGGATGA
GGAGGATGAGGAAGAGGAAGAAGATGAAGAAGATGAGGACGAAGAGGAGGATGATGATGACGAATAA

>RAG1_human
ATGGCTGCTTCATTTCCACCCACATTGGGTTTATCAAGTGCTCCCGACGAAATTCAACACCCACATATAAAATTTTCTGAGTG
GAAATTCAAGTTATTCAGGGTGAGATCCTTTGAGAAAACACCTGAGGAAGCACAAAAGGAAAAAAAGGACAGTTTCGAAGGTA
AGCCATCTTTAGAACAGTCACCCGCAGTCTTAGATAAAGCAGATGGCCAAAAACCAGTTCCCACTCAACCATTGCTAAAGGCT
CATCCAAAATTTAGTAAGAAATTCCACGACAACGAAAAAGCTAGGGGCAAAGCCATACACCAGGCCAATTTAAGGCATTTGTG
CAGAATCTGTGGTAACTCTTTTAGAGCAGACGAGCATAACCGTAGATATCCCGTCCACGGCCCCGTAGACGGAAAAACTTTAG
GACTATTAAGGAAGAAGGAAAAACGTGCCACCTCTTGGCCTGATTTAATCGCTAAAGTGTTCAGGATTGACGTCAAAGCTGAC
GTAGATTCCATCCATCCCACTGAGTTCTGTCATAATTGTTGGTCAATAATGCATCGTAAGTTCTCCTCTGCCCCCTGTGAAGT
ATATTTCCCAAGGAACGTAACAATGGAATGGCACCCACATACACCCTCCTGCGACATATGTAACACCGCAAGAAGAGGATTGA
AAAGAAAATCATTGCAACCCAATTTACAACTAAGTAAGAAGTTAAAGACCGTATTGGATCAAGCCAGGCAGGCTAGACAAAGA
AAGAGACGTGCACAAGCAAGGATCTCCTCTAAAGACGTGATGAAGAAAATAGCTAACTGTTCAAAAATTCACTTATCAACCAA
ATTATTAGCCGTCGATTTCCCAGAGCATTTCGTCAAATCAATATCTTGTCAAATTTGTGAACACATTTTAGCTGATCCAGTAG
AAACCAATTGTAAACATGTCTTTTGTAGGGTTTGCATTTTGAGATGCTTAAAGGTTATGGGTTCTTACTGTCCATCATGCAGA
TACCCATGCTTCCCTACTGATTTGGAAAGTCCTGTGAAGAGTTTTTTGTCTGTCTTGAATTCCCTAATGGTCAAATGCCCTGC
AAAGGAGTGTAATGAAGAGGTATCATTAGAAAAATATAACCATCACATATCCTCACATAAAGAAAGTAAAGAGATCTTCGTGC
ACATTAATAAGGGCGGACGTCCAAGGCAGCATTTGTTGTCTTTAACTAGGAGAGCACAAAAACACAGGTTGAGAGAATTAAAA
TTGCAAGTCAAAGCTTTCGCTGACAAGGAAGAGGGCGGTGATGTTAAGTCAGTTTGTATGACATTATTCTTGTTAGCATTGAG
AGCCAGAAATGAACACAGGCAGGCTGATGAGTTGGAAGCCATAATGCAAGGAAAAGGTTCTGGCCTACAGCCTGCTGTCTGCC
TAGCCATCAGAGTAAATACTTTCTTGTCATGTTCCCAATATCACAAGATGTATAGAACTGTTAAAGCTATCACAGGTAGGCAA
ATTTTTCAACCATTGCATGCATTGAGGAACGCAGAAAAGGTGTTGTTACCAGGTTACCACCACTTTGAATGGCAACCTCCTTT
AAAGAACGTATCTTCCTCTACCGACGTGGGTATAATCGACGGATTGAGTGGCTTGTCATCAAGTGTGGACGACTATCCTGTAG
ATACTATTGCCAAAAGGTTTAGATATGATTCCGCCTTGGTATCTGCATTGATGGACATGGAAGAGGATATCCTAGAGGGTATG
AGGTCACAGGACTTGGATGATTATTTGAATGGTCCTTTCACAGTAGTAGTTAAAGAAAGTTGCGACGGCATGGGCGACGTATC
AGAAAAACATGGTTCCGGACCTGTTGTCCCAGAAAAGGCTGTGAGGTTCTCTTTTACAATCATGAAGATAACAATAGCACATT
CTAGTCAAAATGTAAAGGTGTTTGAAGAAGCTAAGCCAAACTCCGAATTATGTTGCAAGCCTTTATGCCTAATGTTAGCCGAT
GAAAGTGATCACGAAACATTAACCGCTATCTTATCTCCACTAATTGCTGAAAGGGAAGCTATGAAGTCATCAGAGTTAATGTT
GGAGTTGGGTGGTATCTTGAGAACTTTCAAATTCATCTTTAGAGGAACAGGTTACGACGAAAAGTTGGTTAGAGAAGTGGAAG
GTTTAGAAGCTTCCGGATCAGTATATATTTGTACTTTGTGCGATGCAACTAGATTAGAAGCTAGTCAAAACTTAGTCTTTCAT
TCAATAACCAGATCACATGCTGAGAACTTAGAGAGGTATGAAGTATGGAGGTCCAACCCCTATCATGAATCTGTTGAAGAGTT
GAGAGATAGAGTCAAGGGTGTTAGTGCAAAACCTTTCATAGAAACCGTTCCATCCATTGACGCTTTACACTGTGACATAGGTA
ATGCAGCCGAGTTTTACAAGATATTCCAGTTAGAGATAGGTGAGGTATATAAAAATCCAAACGCCTCTAAAGAAGAAAGAAAG
AGATGGCAGGCCACACTAGATAAACACCTAAGAAAGAAGATGAATTTAAAGCCCATCATGAGGATGAACGGTAACTTTGCACG
TAAACTAATGACAAAGGAAACTGTTGACGCCGTATGCGAACTAATCCCTTCCGAAGAAAGGCATGAGGCATTGAGAGAATTGA
TGGACTTGTATTTGAAAATGAAGCCTGTCTGGCGTAGTTCTTGCCCTGCTAAGGAATGTCCAGAATCACTATGTCAGTATTCA
TTCAATTCTCAGAGATTTGCAGAGTTGTTATCCACAAAGTTCAAATATCGTTATGAAGGAAAGATTACAAATTATTTTCACAA
AACATTGGCACATGTCCCAGAAATAATAGAGAGGGATGGTTCCATAGGAGCATGGGCCTCTGAAGGAAACGAATCAGGAAACA
AACTATTCCGTAGATTCAGAAAAATGAACGCAAGACAGAGTAAGTGCTATGAGATGGAAGATGTGCTAAAGCATCACTGGCTA
TATACATCTAAGTACCTACAGAAGTTTATGAACGCCCACAATGCATTGAAAACAAGTGGATTTACCATGAATCCTCAAGCCTC
CTTGGGAGATCCATTGGGTATTGAGGACTCCTTAGAATCTCAAGATTCCATGGAATTCTAA

>RAG2_human

```
ATGAGTCTACAGATGGTCACTGTCTCAAACAATATTGCATTAATTCAGCCTGGATTTTCTTTAATGAATTTCGATGGTCAAGT
TTTTTTCTTCGGCCAGAAAGGTTGGCCTAAGAGATCATGTCCCACCGGAGTCTTCCATTTAGACGTCAAGCATAATCACGTAA
AGTTGAAGCCCACAATTTTCTCCAAGGATAGTTGCTACCTACCTCCACTAAGATATCCTGCCACATGCACCTTCAAAGGATCA
CTAGAGTCTGAAAAACACCAATACATCATTCATGGTGGAAAAACTCCAAATAATGAGGTTTCCGATAAAATTTATGTAATGTC
CATTGTTTGCAAAAACAATAAGAAAGTTACTTTTAGATGTACCGAAAAAGATTTGGTTGGAGACGTGCCAGAGGCAAGATACG
GTCATAGTATTAACGTGGTATACAGTCGTGGCAAAAGTATGGGCGTCTTATTTGGCGGACGTAGTTACATGCCATCCACACAT
AGGACTACCGAAAAGTGGAACAGTGTAGCAGACTGTCTACCTTGCGTATTTTTAGTCGATTTTGAATTCGGATGCGCTACATC
ATACATTTTGCCAGAATTGCAAGACGGTCTAAGTTTTCATGTGTCAATTGCAAAAAATGATACAATTTACATTTTAGGTGGTC
ATTCCTTAGCAAATAATATCAGACCAGCCAATTTGTACCGTATTAGGGTCGATTTACCATTGGGATCACCCGCCGTGAATTGC
ACTGTCTTACCAGGAGGTATCTCTGTATCTTCAGCCATCTTAACTCAGACTAATAATGATGAATTCGTGATAGTAGGTGGCTA
TCAATTAGAAAACCAAAAGAGAATGATTTGTAACATCATTTCCTTGGAGGACAACAAGATTGAAATTAGAGAAATGGAAACCC
CAGATTGGACACCCGATATAAAACACTCAAAGATTTGGTTCGGATCCAACATGGGTAATGGAACTGTTTTCTTGGGAATTCCA
GGCGACAATAAACAAGTGGTTTCTGAAGGTTTTTATTTCTACATGCTAAAGTGCGCAGAAGATGATACCAACGAAGAACAAAC
TACATTCACAAATTCACAGACCTCAACCGAAGACCCAGGCGACTCTACTCCTTTTGAAGACTCAGAAGAGTTTTGCTTTAGTG
CAGAGGCCAATTCCTTTGACGGTGATGACGAGTTCGATACATACAATGAAGACGATGAAGAAGACGAGTCCGAAACTGGCTAT
TGGATAACCTGTTGTCCTACTTGTGATGTTGACATTAATACCTGGGTTCCCTTCTATTCAACTGAGCTAAATAAGCCAGCCAT
GATTTATTGCTCTCATGGCGATGGTCATTGGGTACATGCTCAATGTATGGACTTAGCAGAGAGGACCTTGATTCATTTGTCTG
CCGGTAGTAACAAATATTACTGTAATGAACATGTCGAAATTGCAAGAGCATTACATACACCTCAGAGAGTTTTGCCTTTGAAG
AAACCACCCATGAAATCCTTAAGAAAAAAGGGTAGTGGCAAAATTTTAACTCCTGCTAAAAAATCTTTTTTGAGGCGTTTGTT
TGATTAA
```

Table S3. Recombination target and associated plasmids constructed in this work

Name	Brief Description
pY112-CJA-UP-H20	Split antibiotic resistance recombination, 20-bp homology
pY112-CJA-UP-H20-M12	Split antibiotic resistance recombination, 20-bp homology, mutated 12 RSS
pY112-CJA-UP-H20-M23	Split antibiotic resistance recombination, 20-bp homology, mutated 23-RSS
pY112-CJA-UP-H20-MBoth	Split antibiotic resistance recombination, 20-bp homology, mutated 12- and 23-RSS
pY112-CJA-UP-H20-Double12	Split antibiotic resistance recombination, 20-bp homology, two 12-RSSs
pY112-CJA-UP-H20-Double23	Split antibiotic resistance recombination, 20-bp homology, two 23-RSSs
pY112-CJA-UP-H0	Split antibiotic resistance recombination, 0-bp homology
pY112-CJA-UP-H3	Split antibiotic resistance recombination, 3-bp homology
pY112-CJA-UP-H6	Split antibiotic resistance recombination, 6-bp homology
pY112-CJA-UP-H10	Split antibiotic resistance recombination, 10-bp homology
pY112-CJA-UP-H15	Split antibiotic resistance recombination, 15-bp homology
pY112-CJA-UP-H40	Split antibiotic resistance recombination, 40-bp homology
pY122-CJA-UP-H20	Split antibiotic resistance recombination, 20-bp homology, 2μ ORI (high copy)
pY112-CJA-UPalt-H20	Split antibiotic resistance recombination, 20-bp homology, different split site
pY112-CJA-U-H20	Split antibiotic resistance recombination, 20-bp homology, promoter removed relative to pY112-CJA-UP-H20
pY112-CJA-P-H20	Split antibiotic resistance recombination, 20-bp homology, Ura3 cassette removed relative to pY112-CJA-UP-H20
pY112-CJA-UF-H20	Split antibiotic resistance recombination, 20-bp homology, spacer in pY112-CJA-UP-H20 replaced with URA3 fragment
pY112-CJCG-ES-H20	Split GFP recombination, 20-bp homology, second half fragment order: eGFP then Sapphire
pY112-CJCG-SE-H20	Split GFP recombination, 20-bp homology, second half fragment order: Sapphire then eGFP
pY112-CJCG-EA-H20	Split GFP recombination, 20-bp homology, second half fragment order: eGFP then Azurite
pY112-CJCG-ESA-H20	Split GFP recombination, 20-bp homology, second half fragment order: eGFP then Sapphire then Azurite
pY112-psmTEF1-eGFP	Single-color control for GFP recombination, eGFP
pY112-psmTEF1-Sapphire	Single-color control for GFP recombination, Sapphire
pY112-psmTEF1-Azurite	Single-color control for GFP recombination, Azurite
pY112-SJUE	Signal joint target assay, URA3 and eGFP cassettes flanked by 12- and 23-RSS

pY110-CJCS-PG-H20	Split scFv recombination, 20-bp homology, second half fragment order: PSCA then GPC3
pY110-GPC3	Single-scFv control for antibody recombination, displays anti-GPC3 scFv
pCT-PSCA	Single-scFv control for antibody recombination, displays anti-PSCA scFv

Table S4. RSS sequences

Name	Sequence (5’ heptamer to 3’ nonamer)
12-RSS	CACAGTGCTACAGACTGGAACAAAAACC
23-RSS	CACAGTGGTAGTACTCCACTGTCTGGCTGTACAAAAACC
12-RSS-mut	GTACAGCCTACAGACTGGACAGTCAGTA
23-RSS-mut	GTACAGCGTAGTACTCCACTGTCTGGCTGTCAGTCAGTA
23-RSS-2 (used in split eGFP plasmids)	CACAGTGTGAAAACCCACATCCTGAGAGTGACAAAAACC
23-RSS-3 (used in split eGFP/scFv plasmids)	CACAGTGAGGGGAAGTCATTGTGAGCCCAGACAAAAACC
