# Supplementary material for: Generating combinatorial diversity via engineered V(D)J-like recombination in Saccharomyces cerevisiae
Source: Nat Commun. 2025 Jul 1;16:5688. doi: 10.1038/s41467-025-61206-1 (PMC12216023; doi:10.1038/s41467-025-61206-1)
Supplement: Supplementary file 2 — Reporting Summary [file 41467_2025_61206_MOESM2_ESM.pdf]

nature portfolio

Corresponding author(s): John Blazeck

Last updated by author(s): May 15, 2025

Reporting Summary

Nature Portfolio wishes to improve the reproducibility of the work that we publish. This form provides structure for consistency and transparency in reporting. For further information on Nature Portfolio policies, see our Editorial Policies and the Editorial Policy Checklist.

Statistics

For all statistical analyses, confirm that the following items are present in the figure legend, table legend, main text, or Methods section.

n/a	Confirmed	
☐	☒	The exact sample size (*n*) for each experimental group/condition, given as a discrete number and unit of measurement
☐	☒	A statement on whether measurements were taken from distinct samples or whether the same sample was measured repeatedly
☐	☒	The statistical test(s) used AND whether they are one- or two-sided<br>*Only common tests should be described solely by name; describe more complex techniques in the Methods section.*
☐	☒	A description of all covariates tested
☐	☒	A description of any assumptions or corrections, such as tests of normality and adjustment for multiple comparisons
☐	☒	A full description of the statistical parameters including central tendency (e.g. means) or other basic estimates (e.g. regression coefficient) AND variation (e.g. standard deviation) or associated estimates of uncertainty (e.g. confidence intervals)
☐	☒	For null hypothesis testing, the test statistic (e.g. *F*, *t*, *r*) with confidence intervals, effect sizes, degrees of freedom and *P* value noted<br>*Give P values as exact values whenever suitable.*
☒	☐	For Bayesian analysis, information on the choice of priors and Markov chain Monte Carlo settings
☒	☐	For hierarchical and complex designs, identification of the appropriate level for tests and full reporting of outcomes
☐	☒	Estimates of effect sizes (e.g. Cohen's *d*, Pearson's *r*), indicating how they were calculated

Our web collection on statistics for biologists contains articles on many of the points above.

Software and code

Policy information about availability of computer code

Data collection	Flow cytometry data was collected with BD FACSChorus (for FACSMelody) or Beckman Coulter CytExpert (for Cytoflex). Microscopy images were collected using Zeiss Zen Black software (for Zeiss LSM700).
Data analysis	Plasmids were designed and Sanger sequencing was checked using Geneious Prime 2024.0.3. Flow cytometry data was analyzed using FlowJo 10.10.0. Figures were created and statistical tests were performed using Graphpad Prism 10.3.0. Microscopy images were analyzed with ImageJ 1.54f and Coloc 2 3.1.0 was used to calculate Pearson coefficients.

For manuscripts utilizing custom algorithms or software that are central to the research but not yet described in published literature, software must be made available to editors and reviewers. We strongly encourage code deposition in a community repository (e.g. GitHub). See the Nature Portfolio guidelines for submitting code & software for further information.

Data

Policy information about availability of data

All manuscripts must include a data availability statement. This statement should provide the following information, where applicable:

- Accession codes, unique identifiers, or web links for publicly available datasets
- A description of any restrictions on data availability
- For clinical datasets or third party data, please ensure that the statement adheres to our policy

Annotated target plasmid sequence files are included in the supplementary information. Raw data for all bar charts along with original gel and western blot images are available in the source data file.

Research involving human participants, their data, or biological material

Policy information about studies with human participants or human data. See also policy information about sex, gender (identity/presentation), and sexual orientation and race, ethnicity and racism.

Reporting on sex and gender	*Use the terms sex (biological attribute) and gender (shaped by social and cultural circumstances) carefully in order to avoid confusing both terms. Indicate if findings apply to only one sex or gender; describe whether sex and gender were considered in study design; whether sex and/or gender was determined based on self-reporting or assigned and methods used. Provide in the source data disaggregated sex and gender data, where this information has been collected, and if consent has been obtained for sharing of individual-level data; provide overall numbers in this Reporting Summary. Please state if this information has not been collected. Report sex- and gender-based analyses where performed, justify reasons for lack of sex- and gender-based analysis.*
Reporting on race, ethnicity, or other socially relevant groupings	*Please specify the socially constructed or socially relevant categorization variable(s) used in your manuscript and explain why they were used. Please note that such variables should not be used as proxies for other socially constructed/relevant variables (for example, race or ethnicity should not be used as a proxy for socioeconomic status). Provide clear definitions of the relevant terms used, how they were provided (by the participants/respondents, the researchers, or third parties), and the method(s) used to classify people into the different categories (e.g. self-report, census or administrative data, social media data, etc.) Please provide details about how you controlled for confounding variables in your analyses.*
Population characteristics	*Describe the covariate-relevant population characteristics of the human research participants (e.g. age, genotypic information, past and current diagnosis and treatment categories). If you filled out the behavioural & social sciences study design questions and have nothing to add here, write "See above."*
Recruitment	*Describe how participants were recruited. Outline any potential self-selection bias or other biases that may be present and how these are likely to impact results.*
Ethics oversight	*Identify the organization(s) that approved the study protocol.*

Note that full information on the approval of the study protocol must also be provided in the manuscript.

Field-specific reporting

Please select the one below that is the best fit for your research. If you are not sure, read the appropriate sections before making your selection.

☒ Life sciences ☐ Behavioural & social sciences ☐ Ecological, evolutionary & environmental sciences

For a reference copy of the document with all sections, see nature.com/documents/nr-reporting-summary-flat.pdf

Life sciences study design

All studies must disclose on these points even when the disclosure is negative.

Sample size	No statistical method was used to predetermine sample sizes. In general, data was collected in biological triplicate, which was sufficient to establish statistical significance between different groups.
Data exclusions	No data points were excluded.
Replication	In general, experiments were not repeated to check for reproducibility. However, in certain cases samples were measured under near identical setups in multiple experiments and always showed consistent results.
Randomization	All experiments in this study were done in cells initially derived from a single monoclonal cell line, so no deliberate randomization techniques were warranted.
Blinding	Investigators were not blinded in this experiment. Data was either collected by an instrument (e.g., flow cytometry) or in assays with minimal subjectivity.

Reporting for specific materials, systems and methods

We require information from authors about some types of materials, experimental systems and methods used in many studies. Here, indicate whether each material, system or method listed is relevant to your study. If you are not sure if a list item applies to your research, read the appropriate section before selecting a response.

Materials & experimental systems

n/a	Involved in the study
☐	☒ Antibodies
☐	☒ Eukaryotic cell lines
☒	☐ Palaeontology and archaeology
☒	☐ Animals and other organisms
☒	☐ Clinical data
☒	☐ Dual use research of concern
☒	☐ Plants

Methods

n/a	Involved in the study
☒	☐ ChIP-seq
☐	☒ Flow cytometry
☒	☐ MRI-based neuroimaging

Antibodies

Antibodies used

Anti-myc-AlexaFluor647, Cell Signaling Technology, Cat: 2233S, Clone: 9B11, Lot: 27
Anti-FLAG-APC, BioLegend, Cat: 637308, Clone: L5, Lot:B419567
Anti-RAG2, Invitrogen, Cat: PA5-76764, Polyclonal, Lot: AB4637719
Anti-rabbit_IgG-HRP, Invitrogen, Cat: A16096, Polyclonal, Lot: 94-137-022224
Myc and FLAG antibodies were diluted at 1:100. Anti-RAG2 was diluted at 1:1000 and anti-rabbit_IgG was diluted at 1:2000.

Validation

The anti-myc and anti-FLAG antibodies are validated for flow cytometry by the manufacturer. The anti-RAG2 and anti-rabbit_IgG are both validated for western blot by the manufacturer.

Eukaryotic cell lines

Policy information about cell lines and Sex and Gender in Research

Cell line source(s)

BY4742 - Provided by the Storici Lab, School of Biological Sciences, Georgia Institute of Technology
EBY100 - Provided by Anton Bryskin, Center for Molecular Evolution, Georgia Institute of Technology

Authentication

The cell lines were not authenticated.

Mycoplasma contamination

The cell lines are S. cerevisiae and were therefore not tested for mycoplasma contamination.

Commonly misidentified lines
(See ICLAC register)

No commonly misidentified lines were used.

Plants

Seed stocks

Report on the source of all seed stocks or other plant material used. If applicable, state the seed stock centre and catalogue number. If plant specimens were collected from the field, describe the collection location, date and sampling procedures.

Novel plant genotypes

Describe the methods by which all novel plant genotypes were produced. This includes those generated by transgenic approaches, gene editing, chemical/radiation-based mutagenesis and hybridization. For transgenic lines, describe the transformation method, the number of independent lines analyzed and the generation upon which experiments were performed. For gene-edited lines, describe the editor used, the endogenous sequence targeted for editing, the targeting guide RNA sequence (if applicable) and how the editor was applied.

Authentication

Describe any authentication procedures for each seed stock used or novel genotype generated. Describe any experiments used to assess the effect of a mutation and, where applicable, how potential secondary effects (e.g. second site T-DNA insertions, mosiacism, off-target gene editing) were examined.

Flow Cytometry

Plots

Confirm that:

- [x] The axis labels state the marker and fluorochrome used (e.g. CD4-FITC).
- [x] The axis scales are clearly visible. Include numbers along axes only for bottom left plot of group (a 'group' is an analysis of identical markers).
- [x] All plots are contour plots with outliers or pseudocolor plots.
- [x] A numerical value for number of cells or percentage (with statistics) is provided.

Methodology

Sample preparation	For GFP analysis, approximately 1x10^6 cells were spun down from culture, rinsed with phosphate-buffered saline (PBS), and resuspended in PBS. For antibody surface display, cells were cultured in buffered induction media. 5x10^6 cells were spun down and rinsed with PBS with 0.1% w/v BSA. Cells were then stained with biotinylated antigen for 1 hour, rinsed, then stained with streptavidin-PE and either an anti-myc or anti-FLAG antibody for 30 mins. The cells were rinsed one final time, resuspended in PBS with 0.1% BSA and then analyzed on the flow cytometer.
Instrument	BD FACSMelody or Beckman Coulter CytoFLEX
Software	BD FACSChorus or Beckman Coulter CytExpert were used for collection and FlowJo 10.10.0 was used for analysis.
Cell population abundance	No sorting was performed in this study. Certain cell populations generated during recombination were in low abundance. Therefore 100,000 events were recorded to ensure multiple events were collected represent the population.
Gating strategy	Forward scatter singlets were identified from the initial scatter plot by plotting FSC area versus FSC height and drawing a gate around cells along the main diagonal. All additional gates were drawn on the FSC singlet population. Positive and negative controls were used to draw gates, with gates drawn to assure that >99% of negative cells fell outside of the positive gate. For GFP, FSC area and GFP area were plotted and a single gate was drawn. For eGFP/Sapphire/Azurite comparisons, the channels for eGFP and Sapphire were plotted and gates were drawn to discriminate these two populations. For Azurite, a gate was drawn after channels for Azurite and Sapphire were plotted. For display, APC or AlexaFluor647 Area were plotted versus PE and a quadrant gate was used.

- [x] Tick this box to confirm that a figure exemplifying the gating strategy is provided in the Supplementary Information.
