# Supplementary material for: Generating combinatorial diversity via engineered V(D)J-like recombination in Saccharomyces cerevisiae
Source: Nat Commun. 2025 Jul 1;16:5688. doi: 10.1038/s41467-025-61206-1 (PMC12216023; doi:10.1038/s41467-025-61206-1)
Supplement: Supplementary file 4 — Description of Additional Supplementary Files [file 41467_2025_61206_MOESM4_ESM.pdf]

Supplementary Dataset 1: Target substrate encoding plasmid sequences used for the testing of Homology-assisted recombination.
